# Novel *C16orf57 *mutations in patients with Poikiloderma with Neutropenia: bioinformatic analysis of the protein and predicted effects of all reported mutations

**DOI:** 10.1186/1750-1172-7-7

**Published:** 2012-01-23

**Authors:** Elisa A Colombo, J Fernando Bazan, Gloria Negri, Cristina Gervasini, Nursel H Elcioglu, Deniz Yucelten, Ilknur Altunay, Umram Cetincelik, Anna Teti, Andrea Del Fattore, Matteo Luciani, Spencer K Sullivan, Albert C Yan, Ludovica Volpi, Lidia Larizza

**Affiliations:** 1 Dipartimento di Medicina, Chirurgia e Odontoiatria, Università degli Studi di Milano, Milano, Italy; 2 NeuroScience, Osceola, WI, USA; 3 Istituto di Genetica Medica, Università Cattolica del Sacro Cuore, Roma, Italy; 4 Department of Pediatric Genetics, Marmara University Hospital, Istanbul, Turkey; 5 Department of Dermatology, Marmara University Hospital, Istanbul, Turkey; 6 Sisli Etfal Research and Training Hospital, Department of Dermatology, Istanbul, Turkey; 7 Sisli Etfal Research and Training, Department of Medical Genetics Istanbul, Turkey; 8 University of L'Aquila, Department of Experimental Medicine, L'Aquila, Italy; 9 Ospedale Pediatrico Bambin Gesù, Roma, Italy; 10 OncoHematology Department, Children Hospital Bambino Gesù, Roma, Italy; 11 Children's Hospital of Philadelphia, Philadelphia, PA, USA; 12 Dipartimento di Biologia e Genetica per le Scienze Mediche, Università degli Studi di Milano, Milano, Italy

**Keywords:** Poikiloderma with Neutropenia, Dyskeratosis Congenita, Rothmund-Thomson, *C16orf57*, Founder effect, Bioinformatic prediction of C16orf57 protein, 2H phosphoesterase superfamily, RNA processing, Myelodysplasia

## Abstract

**Background:**

Poikiloderma with Neutropenia (PN) is a rare autosomal recessive genodermatosis caused by *C16orf57 *mutations. To date 17 mutations have been identified in 31 PN patients.

**Results:**

We characterize six PN patients expanding the clinical phenotype of the syndrome and the mutational repertoire of the gene. We detect the two novel *C16orf57 *mutations, c.232C>T and c.265+2T>G, as well as the already reported c.179delC, c.531delA and c.693+1G>T mutations. cDNA analysis evidences the presence of aberrant transcripts, and bioinformatic prediction of C16orf57 protein structure gauges the mutations effects on the folded protein chain.

Computational analysis of the C16orf57 protein shows two conserved H-X-S/T-X tetrapeptide motifs marking the active site of a two-fold pseudosymmetric structure recalling the 2H phosphoesterase superfamily. Based on this model C16orf57 is likely a 2H-active site enzyme functioning in RNA processing, as a presumptive RNA ligase.

According to bioinformatic prediction, all known *C16orf57 *mutations, including the novel mutations herein described, impair the protein structure by either removing one or both tetrapeptide motifs or by destroying the symmetry of the native folding.

Finally, we analyse the geographical distribution of the recurrent mutations that depicts clusters featuring a founder effect.

**Conclusions:**

In cohorts of patients clinically affected by genodermatoses with overlapping symptoms, the molecular screening of *C16orf57 *gene seems the proper way to address the correct diagnosis of PN, enabling the syndrome-specific oncosurveillance.

The bioinformatic prediction of the C16orf57 protein structure denotes a very basic enzymatic function consistent with a housekeeping function. Detection of aberrant transcripts, also in cells from PN patients carrying early truncated mutations, suggests they might be translatable. Tissue-specific sensitivity to the lack of functionally correct protein accounts for the main cutaneous and haematological clinical signs of PN patients.

## Background

Biallelic mutations of the *C16orf57 *(OMIM*613276) gene underlie Poikiloderma with Neutropenia (PN; OMIM#604173), an inherited genodermatosis characterized by early onset poikiloderma, pachyonychia, palmo-plantar hyperkeratosis, skeletal defects and non-cyclic neutropenia. This condition results in recurrent infections during infancy and childhood, primarily of a pulmonary nature, and contributes to the postnatal growth delay in weight and height of the patients.

The syndrome was first described by Clericuzio in Navajo Indians [1] and subsequently in patients of Caucasian ancestry from different geographic areas [2-5]. Following discovery of the causative gene [6] and molecular evidence for distinct genetic control between PN and Rothmund-Thomson syndrome (RTS; OMIM#268400) [7], 31 PN patients have been tested and found to bear 17 different mutations in the responsible *C16orf57 *gene, 84% of the patients were found in the homozygous state [6,8-12] and only six were compound heterozygous [6,11,13]. Interestingly this cohort includes patients previously diagnosed as affected with Dyskeratosis Congenita (DC; OMIM#224230) and with Rothmund-Thomson syndrome illustrating significant phenotypic overlap among these entities [10,13]. All the identified mutations, six nonsense, six frameshifts and five splicing mutations including the apparent missense change, c.502A>G [6], lead to loss-of-function. Despite the limited mutational repertoire a few recurrent mutations have been identified, including c.496delA, common among those of Athabaskan ancestry [11], c.531delA in Turkish families [10,12] and c.179delC in patients of North African origin [8,12], consistent with founder mutations restricted to different geographic areas.

Genotyping further patients could better define the spectrum, the type and the geographical distribution of the *C16orf57 *sequence changes.

The *C16orf57 *gene is phylogenetically conserved and ubiquitously expressed, suggesting a housekeeping function [10].

Whenever cDNA analysis has been performed, mutant alleles have been detected [6,11] hinting they might be translatable. The function of the unidentified C16orf57 protein remains obscure, so we focused on bioinformatic tools to predict the possible structure and ancestry of C16orf57 and to gauge the functional consequences of the different mutations on the folded protein chain, a start point to address genotype-phenotype correlations in the patients.

In this study we characterize six PN patients and define at the DNA and cDNA level the underlying *C16orf57 *mutations. The mutations all present in the homozygous state include a novel early truncating mutation, a novel IVS2 splice-site mutation and an IVS6 splice-site mutation already described but in the heterozygous condition [11].

We also present the computational analysis of the C16orf57 protein chain, showing that two conserved H-X-S/T-X tetrapeptide motifs (where X is a hydrophobic residue) likely mark the active site of a two-fold pseudosymmetric structure related to the 2H phosphoesterase superfamily [14,15]. Based on this analysis we predict that C16orf57 belongs to this enzyme class and the effect of all *C16orf57 *mutations reported so far cause disruptions of both protein fold and catalytic site that hold the critical function of the protein.

## Methods

### Patients

Six patients, three males and three females, referred to us by clinical geneticists and dermatologists, were enrolled in this study. Patients and their parents provided appropriate informed consent.

### DNA extraction and mutation analysis

Genomic DNA was isolated from buccal swabs of patient #26 with NucleoSpin Tissue (Macherey-Nagel, Bethlehem, PA 18020, USA) and from whole peripheral blood of patients #11, #16, #17a, #21, #25 and their relatives using the Wizard Genomic DNA Purification Kit (Promega Corporation, Madison, WI, USA).

About 100 ng of DNA were amplified using GoTaq polymerase (Promega) with previously published primers and conditions [6]. Amplicons were sequenced using Big Dye Terminator v.1.1 Cycle Sequencing Kit according to the manufacture's protocol on the ABI PRISM 3130 sequencer (Applied Biosystems, Foster City, CA, USA). Electropherograms were analyzed with ChromasPro software 1.42 (Technelysium Pty Ltd, Tewantin QLD, Australia) using the wild type sequence of *C16orf57 *gene [ENSG00000103005] as reference.

### RNA extraction and RT-PCR analysis

EBV-transformed lymphoblastoid cell lines (LCLs) were established for patients #21, #17a and their parents. LCLs were cultured in RPMI 1640 medium with 2 mM L-glutamine (EuroClone, Milano, Italy) supplemented with 20% foetal bovine serum (Lonza, Walkersville, MD, USA) and 1% Penicillin, Streptomycin and Ampicillin in 37°C humidified incubator with 5% CO_2_.

Total RNA was extracted from LCLs using TRI-Reagent RNA Isolation reagent (Sigma-Aldrich, Saint-Louis, MI, USA) and from whole blood of patient #26 using PAXgene Blood RNA Kit (PreAnalitix, Hombrechtikon, Swiss).

500 ng of total RNA was reverse-transcribed into cDNA using High Capacity cDNA Reverse Transcription Kit (Applied Biosystems) with random hexamers. PCR amplification of the *C16orf57 *gene transcripts was performed for patients and positive controls using GoTaq polymerase (Promega). Specific primers and conditions for *C16orf57 *transcripts are listed in additional file 1, Table S1. Nucleotide sequences were compared to the major *C16orf57 *transcript reference sequence [ENST00000219281].

### Computational analysis

An evolutionary and structural profile of C16orf57 was constructed by iterative PsiBLAST searches of Genbank [16] followed by PsiPRED secondary structure prediction of the resulting multiple sequence alignment [17]. This alignment was also used by HHrep [18] to look for internally repeated segments of sequence and predicted structure, and by HHpred to locate potentially related folds from the PDB by sensitive HMM-HMM comparison [19]. Comparative modeling of the C16orf57 three-dimensional structure from fold recognition-derived templates was performed by MODELLER [20] and I-TASSER [21]. Surface patterns of conservation and variability in the resulting model were derived by ConSurf [22]. Structure manipulation and electrostatic potential surface visualization of C16orf57 and related structures were done with Pymol http://www.pymol.org.

## Results

### Clinical findings

The features of the six patients with a suspected PN clinical diagnosis are listed in Table 1.

**Table 1:** Clinical features of the investigated PN patients

Patients		#25	#16	#17a	#17b	**#21**[**^23^**]	**#26**[**^24^**]	#11
**Gender**		F	M	M	F	F	M	F

**Date of birth**		2010	2000	1993	1983-1993	1989	1988	1974

**Geographic origin**		US	Turkey	Turkey	Turkey	Italy	Turkey	Algeria

**Clinical signs**	***poikiloderma ***	yes	yes	yes	yes	yes	yes	yes
	
	***onset***	5 months	6 months	5 months	infancy	6 months	6 months	at birth
	
	***first localization***	extremities	extremities	extremities	extremities	feet, hands, knee	widespread	n.d.
	
	***photo-sensitivity***	n.d.	no	no	n.d.	yes	yes	n.d.
	
	***palmo-plantar lesions***	palmo-plantar xerotic erythema	plantar hyper-keratosis desquamation	palmo-plantar hyper-keratosis	palmo-plantar bullous changes	palmo-plantar and knee hyper-keratosis	n.d.	palmo-plantar hyper-keratosis
	
	***nail abnormalities***	multiple nails with distal onycholysis	pachyonychia	toes nails subungueal hyper-keratosis	n.d.	nail dystrophy	digit dystrophy; toes anonychya	big toes
	
	***dental defects***		caries tendency	dental eruption delay; caries tendency	n.d.	dental eruption delay	n.d.	n.d.
	
	***craniofacial dysmorphisms***	*caput quadratum*; prominent forehead	saddle nose	saddle-short nose; flat broad face; *caput quadratum*; retrognathia	n.d.	saddle nose, triangular face	maxillary hypoplasia; saddle nose; micrognathia	n.d.
	
	***skeletal defects***	not apparent at chest X-ray	widening of femoral metaphysis		n.d.	osteopetrosis at 2 m; increased bone density; sclerosis of vertebrae and skull; delayed skeletal maturation	zygodactyly between 2^nd ^and 3^rd ^digit; osteopenia of all bone structures	presence of calcification; multiple bone fractures
	
	***haematological features***	neutropenia	persistent leukopenia	leukopenia; MDS; splenomegaly	n.d.	severe anemia; neutropenia; responsive to GCSF	neutropenia	neutropenia; chronic haemolysis
	
	***recurrent infections***	viral hepatitis (1 month); *S. bovis *bacteremia (3 months)	pneumonia	pneumonia; meningitis	multiple infections	dental abscess; severe sepsis; pneumonia; broncho-pneumonia	recurrent leg ulcers with severe sepsis	infections since infancy
	
	***growth delay***	n.d.	low stature (< 3rd centile)	low stature (< 3rd centile)	n.d.	low stature	low stature	n.d.
	
	***sexual development***	n.d.	normal	hypo-gonadism	n.d.	n.d.	secondary sex features poorly developed; hypo-gonadism	n.d.
	
	***others***	pulmonic stenosis; prolonged QT at ECG; hypoglycemia	n.d.	miscarriage; hyper-keratosis on ear helixes; dry scalp hair	miscarriage; congenital heart disease; died at 10 years	thin hair	n.d.	atrophic polychondritis

n.d. = not detected

The cohort comprises two previously described cases, one Italian girl (#21) with the association of Osteopetrosis with Poikiloderma [23] and one young adult male (#26) from Turkey diagnosed as Rothmund-Thomson [24], and four patients of novel description from North Africa (#11), US (#25) and Turkey (#16, #17a), respectively. The affected sister of case #17a is included as #17b in Table 1 although her clinical signs are partially recorded due to premature death.

**Figure 1:**
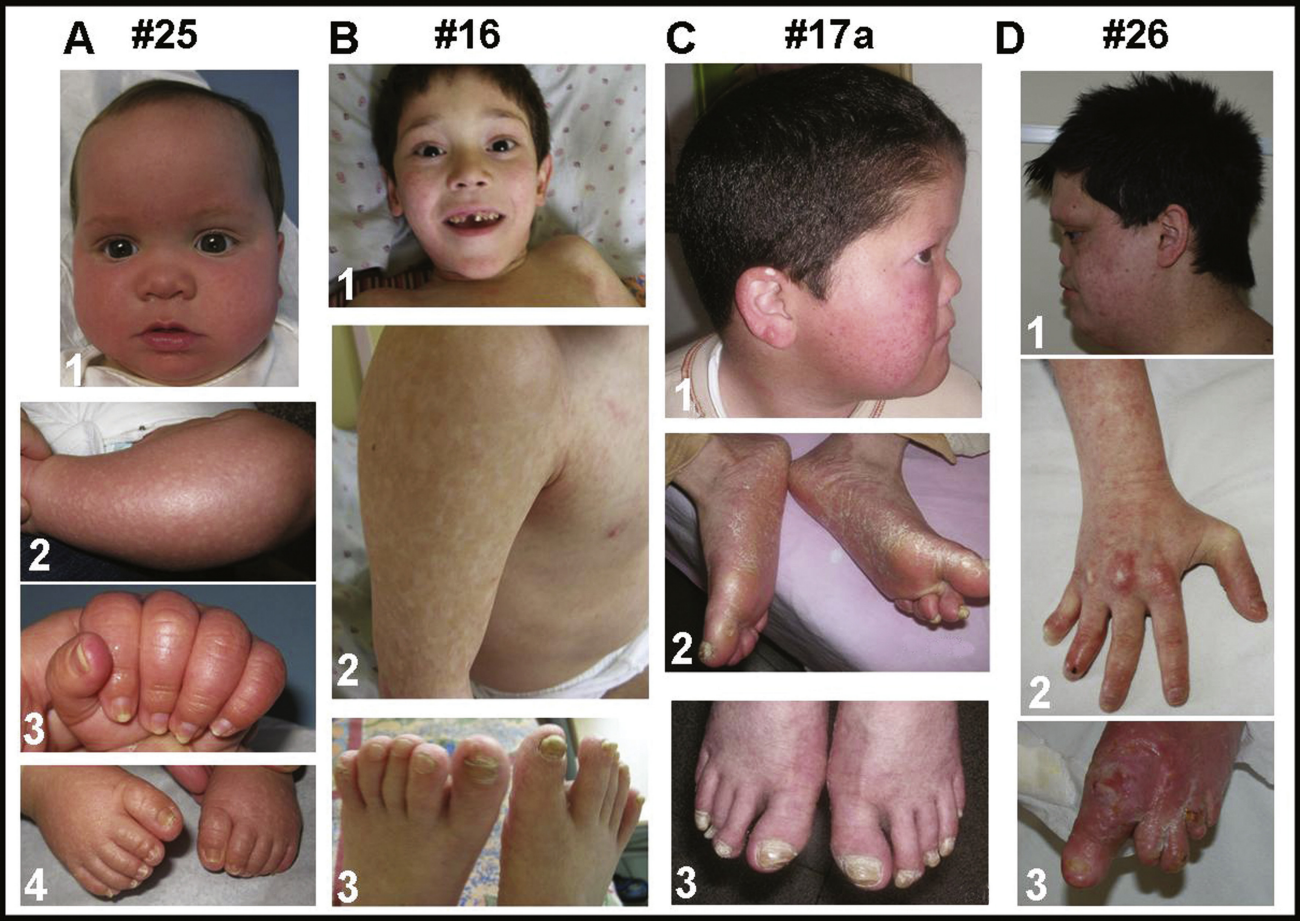
**Clinical findings of four differently aged PN patients**. Panel A refers to the US patient (#25); B, C and D to the Turkish patients #16, #17a and #26, respectively. Patient #25, the youngest in our cohort shows an erythroderma characterized by background erythema and islands of relative sparing on face and legs (A1, A2) and distal onycholysis of fingers and toes (A3, A4). Patient #16 face: poikiloderma and carious teeth are well apparent (B1). Poikiloderma is also visible on the trunk and arm (B2). Pachyonychia of the toes is shown (B3). Facial view of patients #17a and #26 demonstrating prominent forehead, saddle nose and long philtrum (C1, D1); poikiloderma is evident on the face and on the ear helix too (C1) and forearm (D2). Plantar hyperkeratosis (C2) and nail thickening (C3) can be seen. Severe malformation of hands and feet with unhealing ulcers and marked nail dystrophy (D2, D3).

Pictures of four cases are provided in Figure 1, from left to right, according to age ranging from early infancy (#25, panel A), to childhood (#16, panel B), to teen age (#17a, panel C) to young adulthood (#26, panel D).

The acute phase of the rash is visible in the 5 months old infant (#25) who displayed, firstly on arms and legs and subsequently on buttocks and face (Figure 1 A1, A2), flesh-colored papular changes superimposed on background scaly erythema. These early skin findings which have been described at times as ichthyosiform [4] later evolve into a more characteristic chronic poikiloderma visible in all the elder patients (#16, Figure 1 B1, B2; #17a, Figure 1 C1; #26, Figure 1 D1).

As outlined in Table 1 all our patients have nail dystrophy, most often pachyonychia, but anonychia was observed in one case (#26, Figure 1 D2, D3). The nail dystrophy progresses from multiple nails with distal onycholysis involving fingers and toes, as shown in the infant (#25, Figure 1 A3, A4) to clear-cut pachyonychia which is present in the two older male patients (#16, Figure 1 B3; #17a, Figure 1 C2, C3).

Palmo-plantar hyperkeratosis is shared by most cases as well as a tendency to develop dental caries and tooth eruption delay (Table 1; Figure 1 B1, C2).

Supplemental skin findings include thin hair (hypotrichosis in #21) and actinic-hyperkeratosis of the helix (#17a).

Craniofacial dysmorphisms include frontal bossing, saddle nose deformity, midfacial hypoplasia, and retrognathia (Table 1; Figure 1 A1, B1, C1, D1).

Bone alterations (Table 1), detectable on radiograph [23,24], include osteopenia (#26), severe toe and fingers dysplasia (#26, Figure 1 D2, D3), osteopetrosis (#21), calcification and multiple fractures in the long bones (#11). Delayed skeletal maturation (#21) and small stature (#16, #17a, #21, #26) are also observed.

Laboratory findings highlight severe, persistent and non-cyclic neutropenia, leading to recurrent respiratory infections, which are sometimes resistant to standard antibiotic treatments. Myelodysplasia and splenomegaly developed in patient #17a. With regard to sexual development, two Turkish males (#17a, #26) presented with hypogonadism. Cardiac abnormalities were observed in case #25 and demonstrated both structural (pulmonic stenosis) and functional abnormalities (ECG alterations). The family history of case #17a included a miscarriage and a sibling (#17b) born with a congenital heart defect.

### *C16orf57 *mutations and transcript analysis

**Figure 2:**
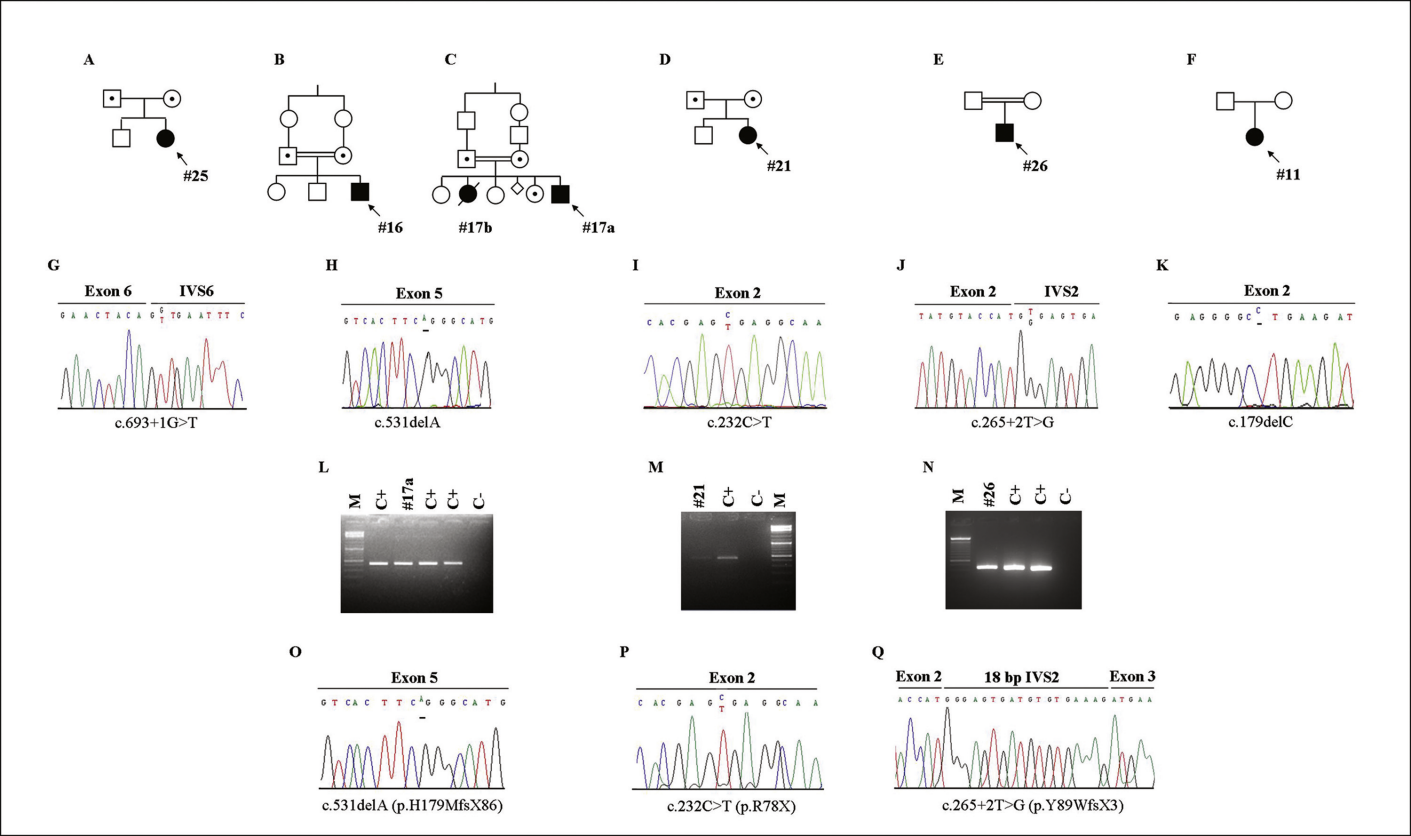
**Pedigrees of index cases and genomic and cDNA characterisation of their *C16orf57 *homozygous mutations**. Pedigrees of patients #25 (A), #16 (B), #17a (C), #21 (D), #26 (E) and #11 (F). Arrows indicate index cases. Direct sequencing of genomic DNA shows homozygous mutations in all cases: c.693+1G>T affecting the IVS6 donor splice site in patient #25 (G), c.531delA in both patients #16 and #17a (H), nonsense c.232C>T in patient #21 (I), c.265+2T>G affecting the IVS2 donor splice site in patient #26 (J) and c.179delC in patient #11 (K). L, M, N show RT-PCR products from patients #17a, #21 and #26, respectively. C+ indicates the positive control with the cDNA source from a healthy individual; C- indicates the negative control with no cDNA added to the reaction; M indicates the molecular weight markers (Generuler DNA ladder mix 100 bp-Fermentas). O, P,Q the corresponding sequencing of mutant transcripts.

The pedigrees of our six PN patients are depicted in Figure 2 (A, B, C, D, E and 2F): the three patients of Turkish ancestry are born to consanguineous parents.

The splicing mutation affecting the donor splice site of IVS6, c.693+1G>T, is detected in homozygous state in the US infant (#25, Figure 2G).

The two Turkish males, #16 and #17a, carry the same homozygous deletion c.531delA within exon 5 (Figure 2H). cDNA amplification showed a PCR product of the expected size and its sequencing confirmed the frameshift mutation c.531delA predicting the aberrant protein p.H179MfsX86 (Figure 2 L, O).

The Italian girl #21 carries the novel homozygous transition c.232C>T within exon 2 (Figure 2I). Direct sequencing of the amplified cDNA fragment revealed the c.232C>T change leading to a premature stop codon p.R78X (Figure 2 M, P).

The third Turkish patient #26 shows the novel homozygous c.265+2T>G transversion affecting the donor splice site of IVS2 (Figure 2J). Direct sequencing of the cDNA fragment evidenced 18 intronic nucleotides added to exon 2 following the activation of a new cryptic splice site in IVS2 and predicting a premature stop codon p.Y89WfsX3 (Figure 2 N, Q).

The c.179delC mutation in exon 2 consistent with the aberrant truncated protein p.P60LfsX54 [8,10] has been identified in the Algerian female patient (#11; Figure 2K).

Additional file 2 Table S2 sums up the location within the gene, the type of mutation and the predicted effect on the C16orf57 protein of the five homozygous mutations detected in the six investigated patients.

### Recurrence and geographic distribution of *C16orf57* mutations

Four (#25, #16, #17a, #11) of our six patients were found to carry previously reported *C16orf57 *mutations [8,10,11].

**Figure 3:**
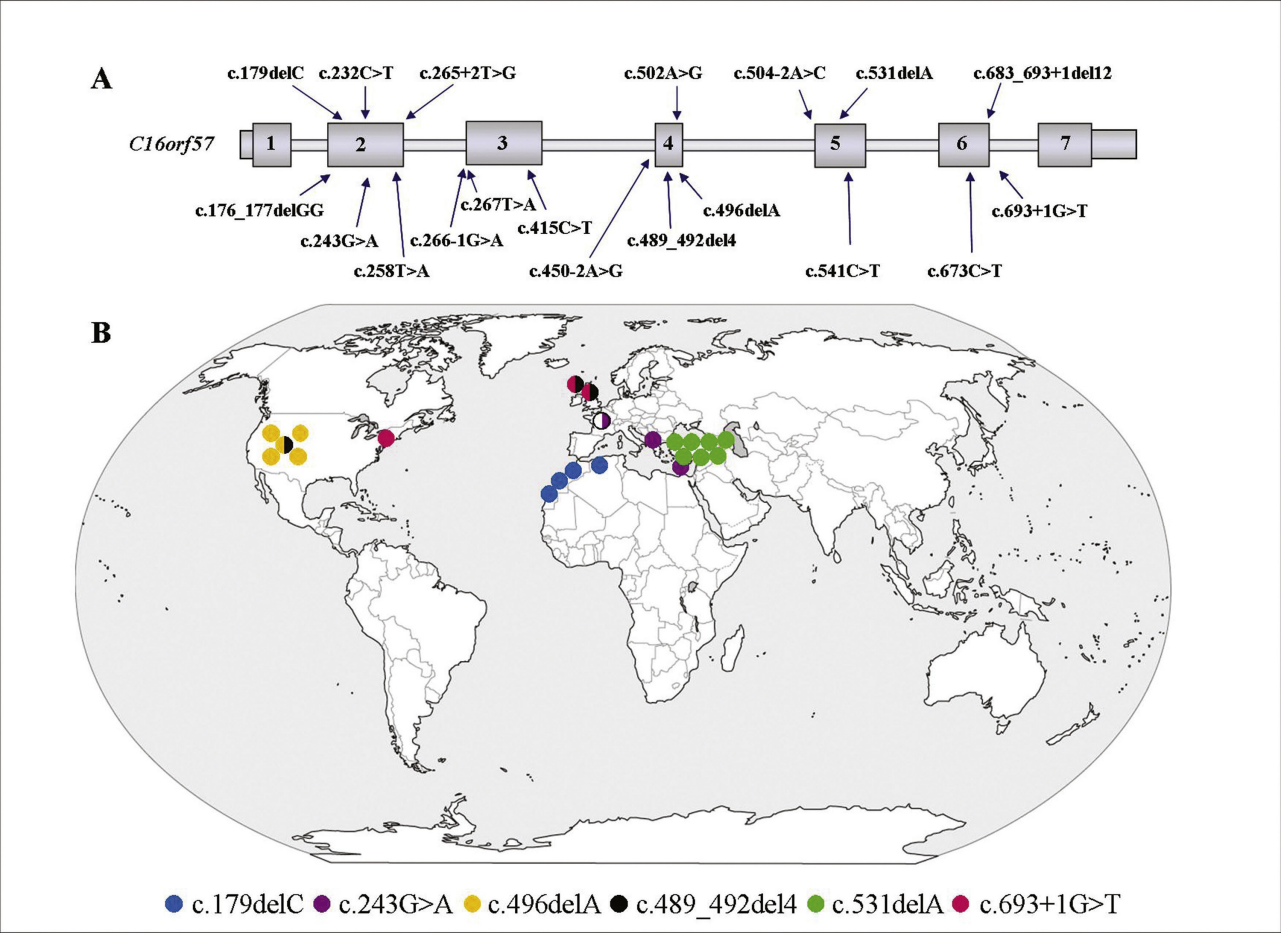
**Overview of recurrent *C16orf57 *mutations**. A) Schematic representation of the *C16orf57 *gene with all sequence alterations so far identified. B) World map of six recurrent *C16orf57 *mutations with geographical distribution. Each bullet represents one tested PN patient. A specific colour is assigned to every mutant allele. Bicolour bullets highlight compound heterozygous patients.

A general overview of all 19 *C16orf57 *mutations detected so far in 37 molecularly tested PN patients depicts all recurrent mutations and their geographic distribution (Figure 3 A, B); 31 out of 37 patients carry mutations in homozygous state (84%) and only six are compound heterozygous.

The most frequent recurrent c.531delA mutation is recorded in the homozygous state in seven patients, including our patients #16 and #17a from two apparently unrelated Turkish families [12] which originate from the same rural area. This deletion has been also reported in five patients of three different families featuring a cluster of Caucasian ethnicity [10] (Figure 3B). Three more PN patients are of Turkish ancestry [3,25].

The second most frequent mutation c.496delA is identified in five patients, four homozygous and one heterozygous, from the Athabaskan inbred ethnic group [11].

Our eldest patient of Algerian origin shares the homozygous mutation c.179delC with a described Moroccan kindred depicting a North African origin of this mutation [4], while the youngest patient of our cohort, from the US, shares the mutation c.693+1G>T with two fraternal twins of Scottish origin [11] (Figure 3B).

Three other mutations have been described in unrelated patients: the early truncating c.243G>A mutation in two Mediterranean families in homozygous state [9,10,26] and in a patient recently reported by a French group in the heterozygous state [13] (Figure 3B); the nonsense c.541C>T mutation reported in two unrelated homozygous patients [10,11] and c.489_492del4 reported in the heterozygous state in one Navajo/Caucasian and two Caucasian sibs [11] (Figure 3B).

### Bioinformatic prediction of the structure of C16orf57 protein

**Figure 4:**
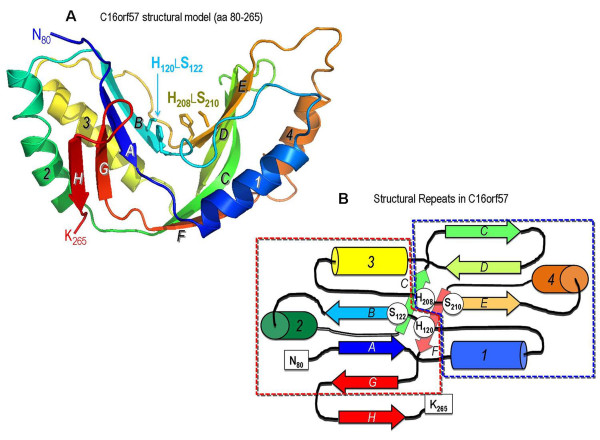
**Structural model of C16orf57 protein**. A) Predicted 2H phosphoesterase family fold of human C16orf57, built by MODELLER [20] from the 1VGJ template structure (HHpred match probability of 99.9, E-value 2.9×10^-29^). Cartoon form with the chain colour-ramped from N-terminal residue 80 (dark blue) to C-terminal residue 265 (red); β-strands are labelled A-H and α-helices numbered 1-4. B) The two α+β lobes form an active site groove marked by the signature 2H motifs. Side chains are shown for the catalytic H_120_xS_122 _and H_208_xS_210 _residues. The flattened chain topology of human C16orf57 shows the structural repeats (boxed) and active site motifs that characterize the 2H phosphoesterase fold family. Identical labels and colours are drawn from the structural model in A and B.

The unidentified C16orf57 protein sequence lacks revealing motifs or homologs through database searches and does not disclose any internal domains by SMART analysis [27]. As protein structure is better conserved than sequence, we focused on more sensitive fold recognition and structure prediction tools [28] as a way to find a fold and related biochemical function for C16orf57. Iterative PsiBLAST searches [16] were first used to assemble a phylogenetically diverse set of C16orf57 paralogs from Genbank. Subsequently, a PsiPRED-derived secondary structure profile of the resulting alignment [17] suggested the presence of a α+β-rich domain in the C-terminal 185 residues of the C16orf57 chain. Within this conserved domain, the HHrep web server [18] detected a faint but significant duplication of sequence and secondary structure patterns, prominently anchored by conserved H-X-T/S-X tetramotifs that correspond to **H_120_**L**S_122_**L and **H_208_**L**S_210_**L sequences in human C16orf57 (Figure 4A). Accordingly, HMM-HMM comparisons by HHpred [19] located a string of significant matches between the C16orf57 domain (residues 80-265) and diverse members of the 2H phosphoesterase fold superfamily that are characterized by a pseudo-two-fold symmetric α+β fold with a central, dual histidines (2H) active site [14].

The implication from these fold recognition results is that C16orf57 is likely a 2H-active site enzyme (Additional file 3 Figure S1) functioning in RNA processing pathways, but the low degree of sequence identity (8-14%) in the HHpred alignments do not unambiguously cluster C16orf57 into one of the four major 2H phosphoesterase families: bacterial 2'-5' RNA ligases, fungal RNA ligases, plant and yeast 1',2'-cyclic nucleotide phosphodiesterases, or mammalian 2',3'-cyclic nucleotide phosphodiesterases (CNPase) [14,29,30]. Still, the highest scoring structural matches to C16orf57 are to: a *Pyrococcus horikoshii *2'-5' RNA ligase (PDB file 1VJG) [29], a central domain from human AKAP18 with a 2H active site (PDB file 2VFK) [30] that binds AMP, lacks CNPase activity and resembles bacterial RNA ligase structures; and the *Bacillus subtilis *YjcG protein, a putative 2'-5' RNA ligase (PDB file 2D4G) [31].

To test the structure-based assertion that C16orf57 is a presumptive RNA ligase, the human C16orf57 2H phosphoesterase fold was modelled with the top HHpred match (1VJG) as the template for MODELLER [20], and also by I-TASSER drawing from multiple templates [21]. The resulting comparative models are quite similar (1.42 Å RMSD over 177 aligned C_α _positions), with differences parcelled out to loops and short helices. The characteristic geometry of the 2H active site is preserved in C16orf57, with catalytic **H_120 _**and **H_208 _**residues poised across an active site cleft between the two symmetric α+β lobes (Additional file 3 Figure S1). Drawing from the common catalytic mechanism of 2H phosphoesterase structures [29,30], these enzymes are involved in the hydrolysis of 2',3'- or 1',2'-cyclic phosphates to 2'- or 1'-phosphates. The histidine residues signature functions either as a nucleophilic attacking group for the cyclic phosphate (C-terminal His), or protonate the leaving oxygen in the reaction (N-terminal His). In bacteria, the 2'-5' RNA ligase acts to ligate half-tRNA molecules (one half with a 2',3'-cyclic phosphate, the other half presenting a 5'-OH terminal). However, similar to the case of the RNA-ligase-like structure of the human AKAP18 2H domain [30], the true substrates of the C16orf57 enzyme remain unknown and await experimental elucidation.

### Predicted effects of all described *C16orf57 *mutations according to the 3D model

**Figure 5:**
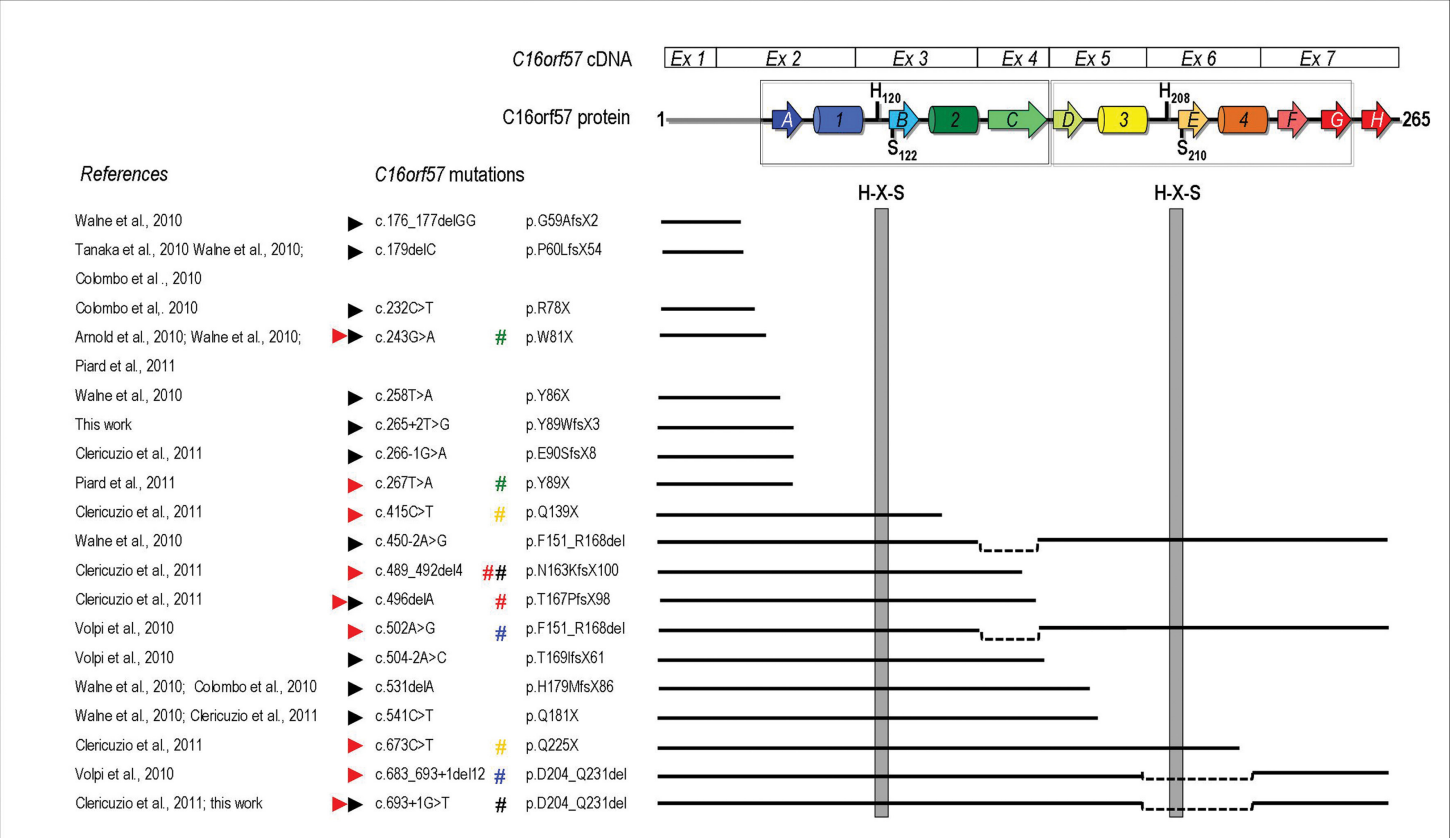
**Structural implications of *C16orf57 *mutations in PN patients**. Predicted disruption of protein structure caused by 19 *C16orf57 *mutations (references in the first column). The N- and C-terminal sequence repeats detected by HHrep are encoded by similar exon arrays (exons 2-4 and 5-7, respectively). The correspondence between gene exons and protein domains (using the topology map of Figure 4 with similar colours and labels) is pointed out focusing on the two H-X-S motifs (grey vertical bars) that form the C16orf57 catalytic site. The top eight mutations lead to loss of both H-X-S motifs as they predict early truncation by a stop codon (c.232C>T, c.243G>A, c.258T>A and c.267T>A), frameshift (c.176_177delGG, c.179delC) or missplicing leading to frameshift (c.265+2T>G, c.266-1G>A). Six subsequent mutations terminate the protein chain before the second H-X-S motif: they include nonsense c.415C>T and c.541C>T, frameshift c.489_492del4, c.496delA and c.531delA and splice site mutation c.504-2A>C. Two mutations lead to the loss of the second H-X-S domain by inframe exon 6 skipping caused by frameshift, c.683_693+1del12, or missplicing, c.693+1G>T. Splicing c.450-2A>G and c.502A>G mutations should maintain both the key motifs, but due to inframe exon 4 skipping the protein loses a critical structural element and likely can not fold properly. Lastly, the c.673C>T stop mutation predicts a shorter chain endowed with both catalytic motifs, but unable to complete the active structure. Prediction of the effects of the different mutations is made more complex by the homozygous versus the heterozygous state. Black arrowheads indicate mutations found in the homozygous state while red arrowheads those found in the heterozygous state; the colour-code of the # symbol is according to the partnership.

The 2H phosphoesterase fold of human C16orf57 is particularly sensitive to the effects of mutations that cause deletions or truncations in the protein chain because of the nature of the internal sequence repeats that are interdigitated between the two structural repeats or lobes (Figure 4B). These lobes frame the active site groove and position the two catalytic His residues, but since the chain topology swaps β-strands and α-helices between lobes, the linear sequence repeats are individually incapable of forming a well-folded lobe-like domain. Since most of the *C16orf57 *mutations catalogued in the present study cause premature terminations of the protein chain (Figure 5), the resulting protein fragment is either comprised of (a) part of the N-terminal sequence repeat, (b) exactly the N-terminal repeat or (c) the complete N-terminal repeat plus some part of the C-terminal repeat. In all three cases, the mutant protein is unlikely to fold into a well-structured lobe; in addition, the frequent loss of the C-terminal nucleophilic His (Figure 5) further ensures the destruction of the 2H active site. The two instances of deletions of a protein segment that encompasses β-strand C (in the N-terminal sequence repeat) would also be severely disruptive to proper folding of an active protein (Figure 4B).

## Discussion

Since the discovery of the causative gene, Poikiloderma with Neutropenia syndrome can now be confirmed by molecular diagnostic testing for mutations in the *C16orf57 *gene and thereby differentiated from the phenotypically similar clinical entities Rothmund-Thomson syndrome and Dyskeratosis Congenita. Based on the molecular test, patients previously diagnosed on clinical grounds as having RTS or DC have been successfully reclassified [13].

We have identified biallelic mutations of the *C16orf57 *gene in six patients including #26, previously described as atypical Rothmund-Thomson [24] and #21, reported as affected with Poikiloderma associated with Osteopetrosis [23]. All the patients herein molecularly confirmed as PN display the consensus PN clinical signs, i.e. early-onset poikiloderma involving the extremities and extending to the face, pachyonychia, palmo-plantar hyperkeratosis and non-cyclic neutropenia (Table 1). It is worth mentioning that our patient #17a shows poikiloderma also on the ear helix, as previously reported in other patients [3,9].

Craniofacial dysmorphisms have been reported only in a few PN patients [5,32]; facial features such as a saddle nose and midfacial hypoplasia are displayed by four out of our six patients, suggesting they are quite common and hence should be recorded during PN clinical evaluation. Dental defects are common in our cases, in agreement with the literature [4,13,26,33]. Hypogonadism was identified in male patients #17a and #26 and previously reported [3,34]. In contrast to *RECQL4*-positive RTS, where bony changes constitute a major diagnostic sign [7], skeletal involvement has only seldom been described in PN patients [3,13,26]. Indeed, apart from our patients #17a and the infant #25, all herein described PN patients display overt skeletal signs including zygodactyly between the second and third digit (#26), multiple bone fractures (#11), intermediate osteopetrosis (#21) or X-ray detectable skeletal findings (#16) (Table 1). A relationship may be envisaged between zygodactyly and the swan neck hand hyperflexibility noticed in a few described patients [3,5,33]. More generally delayed bone maturation has been recorded [32] in the girl subsequently confirmed to carry two distinct *C16orf57 *mutations [6], and diffuse osteosclerosis has been underlined by Porter [26] in the patient found to harbour *C16orf57 *mutations [10]. This patient is one of three cases reported as RTS [10] with defects in the bone marrow, as reported for other clinically diagnosed RTS cases [32-34]. We believe these cases, previously classified as affected by RTS, more correctly represent cases of PN as confirmed by molecular testing of some of them [6,10]. All our patients display non-cyclic neutropenia, the hallmark of PN, and this is a fundamental sign differentiating PN from both RTS or DC, which should be searched also in suspected cases where it remains silent [13]. Neutropenia leads to recurrent infections, which are widely documented in the paediatric histories of PN cases and confers upon them a high risk to develop myelodysplasia from the second decade of life, as attested in at least ten *C16orf57-*positive patients [5,10] and our patient #17a. When considering bone marrow hypocellularity, increased myeloid precursors and delayed neutrophils maturation, a higher percentage of *C16orf57*-positive cases displays this feature [4,5,10,11] highlighting the sensitivity of the myeloid lineage to *C16orf57 *mutations. Evolution to acute myeloid leukemia has been reported in a few PN patients [10,26] and in other untested cases [25,33-35]. This evidence features PN as a cancer predisposing syndrome affecting the myeloid compartment and connects PN to RTS, a syndrome with an increased risk for osteosarcoma and skin cancer [7], and DC which predisposes to a wide variety of haematological and solid tumors [36]. Molecular characterization is thus compulsory for assigning appropriate oncological surveillance.

As depicted in Figure 3 the recurrence of the c.531delA, c.496delA and c.179delC mutations delineates three clusters according to the geographic origin of the patients. A common ancestor is the likely hypothesis to explain the recurrence of these mutations restricted to specific ethnic groups, considering the very low frequency of the PN syndrome and the prevalence of patients with homozygous mutations (31 out of 37). Genetic analysis and reconstruction of ancient genetic links through haplotype segregation analysis could confirm this assumption.

Expansion of the *C16orf57 *mutational repertoire and validation of the observed geographical distribution may allow assessment of clinical variability of PN phenotype in distinct founder mutation cohorts, as it has been described for another autosomal recessive developmental disorder [37].

Lack of information of the function of C16orf57 protein makes it difficult to establish a link between mutations and the onset and evolution of syndromic presentation.

Bioinformatic analysis can provide a preliminary tool to predict the severity of specific mutations.

The structure-based inference that human C16orf57 is a member of the 2H phosphoesterase superfamily, despite very little sequence identity, rests on the recognition of a common protein fold by sensitive algorithms that weave together evolutionary information, in the form of sequence patterns that are conserved across C16orf57 orthologs, and accurate secondary structure predictions to comprehensively scan structural databases. The most prominent pattern in C16orf57 is a two-fold repeated sequence and structural segment with signature H-X-T/S-X tetramotifs; these conserved features are precisely mirrored in the fold-recognition-derived matches with bilobal 2H phosphoesterase folds that rely on the symmetrically poised His residues for catalytic activity. The 2H phosphoesterase superfamily is a diverse grouping of enzymes with a common core architecture, a basic hydrolytic focus for cyclic phosphates, and some functional variability in both substrates and reactions [14,15]. This degree of functional diversification is observed in other structurally-assembled superfamilies of enzymes [38]. Of the various biological tasks performed by 2H active site enzymes, we argue (by closer structural resemblance) that C16orf57 is perhaps an RNA ligase though the actual targets or substrates of this activity remain unknown.

All the mutations reported in our patients are in the homozygous state, which facilitates transcript analysis and understanding the effect of the mutations.

Indeed with the exception of the Algerian patient #11, transcripts have been tested, detected and sequenced in all other patients (Figure 2). With regards to the c.693+1G>T mutation in patient #25, transcript analysis has been reported [11] suggesting exon 6 skipping according to the size of the aberrant band; indeed a misspliced 84 base shorter transcript was found associated with the c.683_693+1del12 which affects the same IVS6 donor splice site [6]. The general emerging feature is that the aberrant transcripts are detectable even for early truncating mutations, such as those of patients #21 and #26, pointing out they are relatively stable and translatable.

## Conclusions

It is known that Dyskeratosis Congenita, Rothmund-Thomson and Poikiloderma with Neutropenia have many overlapping features. Starting from the clinical presentation it is very difficult to assess the correct diagnosis, and in fact most of the molecularly confirmed PN patients have a long history of wrong diagnosis, as DC or RTS. The availability of *C16orf57 *molecular testing allows the correct diagnosis which is compulsory for retargeting syndrome-specific oncosurveillance.

As the present study shows, all the 19 *C16orf57 *mutations linked to disease involve the predicted enzymatic domain of C16orf57 protein. By destroying the native fold, all mutations should cause a drastic loss of enzymatic activity. Future studies of protein presence and activity in PN patients could confirm whether and how the aberrant transcripts, which have been always detected whenever assayed, may be translated.

The *C16orf57 *gene is ubiquitously expressed [6,10], but not all tissues are equally affected by the lack of correctly functioning C16orf57 protein during development and throughout life.

The onset of the poikiloderma, nail dystrophy and teeth malformations at early infancy reflects the perturbed morphogenesis of skin and cutaneous annexes, while neutropenia results from impaired homeostasis of the highly C16orf57 expressing myeloid cells [6,10]. The life long risk of myelodysplastic syndrome features the increased tendency to apoptosis and leukemic transformation of C16orf57-defective myeloid progenitor cells [39].

However, whether different mutations impact differently on the clinical phenotype and on the risk of myelodysplastic syndrome awaits further clinical and molecular characterization of PN patients, along with delineation of patients' clinical expressivity in distinct geographical areas and dissection of the biological function of the C16orf57 protein.

## List of abbreviations

PN: Poikiloderma with Neutropenia; RTS: Rothmund-Thomson syndrome; DC: Dyskeratosis Congenita; LCLs: EBV-transformed lymphoblastoid cell lines; 2H: dual Histidines.

## Competing interests

The authors declare that they have no competing interests.

## Authors' contributions

EAC performed molecular analysis and interpreted the predicted effects of mutations, drafted the manuscript and approved the final version. JFB performed and interpreted computational analysis, drafted and revised the article, approved the final manuscript. GN performed and interpreted molecular analysis, mined the literature and approved the final manuscript. CG supported the molecular work and software access. NE and DY contributed with clinical data and biological samples of patients#16 and #17a, provided helpful feedback and approved the final manuscript. NA and UC contributed with clinical data and sample of patient #26 and approved the final manuscript. AT, ADF and ML contributed with clinical data and sample of patient #21 and approved the final manuscript. SKS and ACY contributed with clinical data and sample of patient #25, revised and approved the manuscript. LV contributed to recruit patients, designed the geographical analysis, drafted, revised and approved the manuscript. LL designed and coordinated the study; drafted, revised and approved the manuscript.

## Supplementary Material

Additional file 1 **Primer sequences, amplicons size, annealing temperatures (Ta) for *C16orf57 *cDNA analysis (ENST00000219281 Ensembl database) and analysed patients**. Table S1 provides technical information on PCR conditions used to analyze cDNA of PN patients #17a, #21 and #26. Click here for file

Additional file 2 ***C16orf57 *homozygous mutations in the set of investigated PN patients**. Table S2 provides a summary of identified mutations of PN patients including location within the gene, mutation type and predicted effect on the protein. All mutations are in the homozygous state. Click here for file

Additional file 3 **Electrostatic potential surface of human C16orf57 predicted protein**. The solvent-accessible surface of the I-TASSER-derived [21] structural model of human C16orf57 was displayed in Pymol http://www.pymol.org and coloured according to the electrostatic potential (ESP) ranging from blue (positively charged or basic) to red (negatively charged or acidic). In a top view that looks directly down at the active site groove, the corresponding ESP surface is quite negatively charged, which is similar in nature to 2H phosphoesterase structures of the RNA ligase class that interact with positively charged substrates [29,30]. Click here for file
