# Supplementary material for: Novel C16orf57 mutations in patients with Poikiloderma with Neutropenia: bioinformatic analysis of the protein and predicted effects of all reported mutations
Source: Orphanet J Rare Dis. 2012 Jan 23;7:7. doi: 10.1186/1750-1172-7-7 (PMC3315733; doi:10.1186/1750-1172-7-7)
Supplement: Additional file 1 — Primer sequences, amplicons size, annealing temperatures (Ta) for C16orf57 cDNA analysis (ENST00000219281 Ensembl database) and analysed patients. Table S1 provides technical information on PCR conditions used to analyze cDNA of PN patients #17a, #21 and #26. [file 1750-1172-7-7-S1.PDF]

Additional file 1

Table S1

Title: Primer sequences, amplicons size, annealing temperatures (Ta) for *C16orf57* cDNA analysis (ENST00000219281 Ensembl database) and analysed patients

Description: Table S1 provides technical information on PCR conditions used to analyze *C16orf57* cDNA of PN patients #17a, #21 and #26

Primer name	Primer sequences	Amplicon length	Ta	Patients analysed
F2 (ex3-4)<br>R2 (ex7)	5'-CCTCCTTCCACAGATTCTTC-3'<br>5'-GTTCCTCCATCTCAGCCTG-3'	450 bp	58°C	#17a
F1 (ex1)<br>Ex3R (ex3)	5'- CGGTTGAGGTTGCTGGTGG-3'<br>5'-GGGACAGGCTGAGGTGGAAC-3'	498 bp	58°C	#21, #26
