# Supplementary material for: Novel C16orf57 mutations in patients with Poikiloderma with Neutropenia: bioinformatic analysis of the protein and predicted effects of all reported mutations
Source: Orphanet J Rare Dis. 2012 Jan 23;7:7. doi: 10.1186/1750-1172-7-7 (PMC3315733; doi:10.1186/1750-1172-7-7)
Supplement: Additional file 2 — C16orf57 homozygous mutations in the set of investigated PN patients. Table S2 provides a summary of identified mutations of PN patients including location within the gene, mutation type and predicted effect on the protein. All mutations are in the homozygous state. [file 1750-1172-7-7-S2.PDF]

Additional file 2

Table S2

Title: *C16orf57* homozygous mutations in the set of investigated PN patients

Description: Table S2 provides a summary of identified mutations of PN patients including location within *C16orf57* gene, mutation type and predicted effect on the protein. All mutations are in the homozygous state.

	Mutation			
**Patient**	**Location**	**c.DNA**	**Mutation Type**	**Protein**
#11	Exon 2	c.179delC[a]	Deletion/Frameshift	p.P60LfsX54
#21	Exon 2	c.232C>T	Nonsense	p.R78X
#26	IVS2	c.265+2T>G	Splice site (donor)	p.Y89WfsX3
#16	Exon 5	c.531delA[b]	Deletion/Frameshift	p.H179MfsX86
#17a	Exon 5	c.531delA[b]	Deletion/Frameshift	p.H179MfsX86
#25	IVS6	c.693+1G>T[c]	Splice site (donor)	p.Y89WfsX3

[a] Previously reported [8; 10];
[b] Previously reported [10];
[c] Previously reported in the heterozygous state [11].
