# Supplementary material for: Novel C16orf57 mutations in patients with Poikiloderma with Neutropenia: bioinformatic analysis of the protein and predicted effects of all reported mutations
Source: Orphanet J Rare Dis. 2012 Jan 23;7:7. doi: 10.1186/1750-1172-7-7 (PMC3315733; doi:10.1186/1750-1172-7-7)

Additional file 1

Figure S1

Title: Electrostatic potential surface of human C16orf57 predicted protein

Description: The solvent-accessible surface of the I-TASSER-derived [21] structural model of human C16orf57 was displayed in Pymol (www.pymol.org) and coloured according to the electrostatic potential (ESP) ranging from blue (positively charged or basic) to red (negatively charged or acidic). In a top view that looks directly down at the active site groove, the corresponding ESP surface is quite negatively charged, which is similar in nature to 2H phosphoesterase structures of the RNA ligase class that interact with positively charged substrates [29; 30].

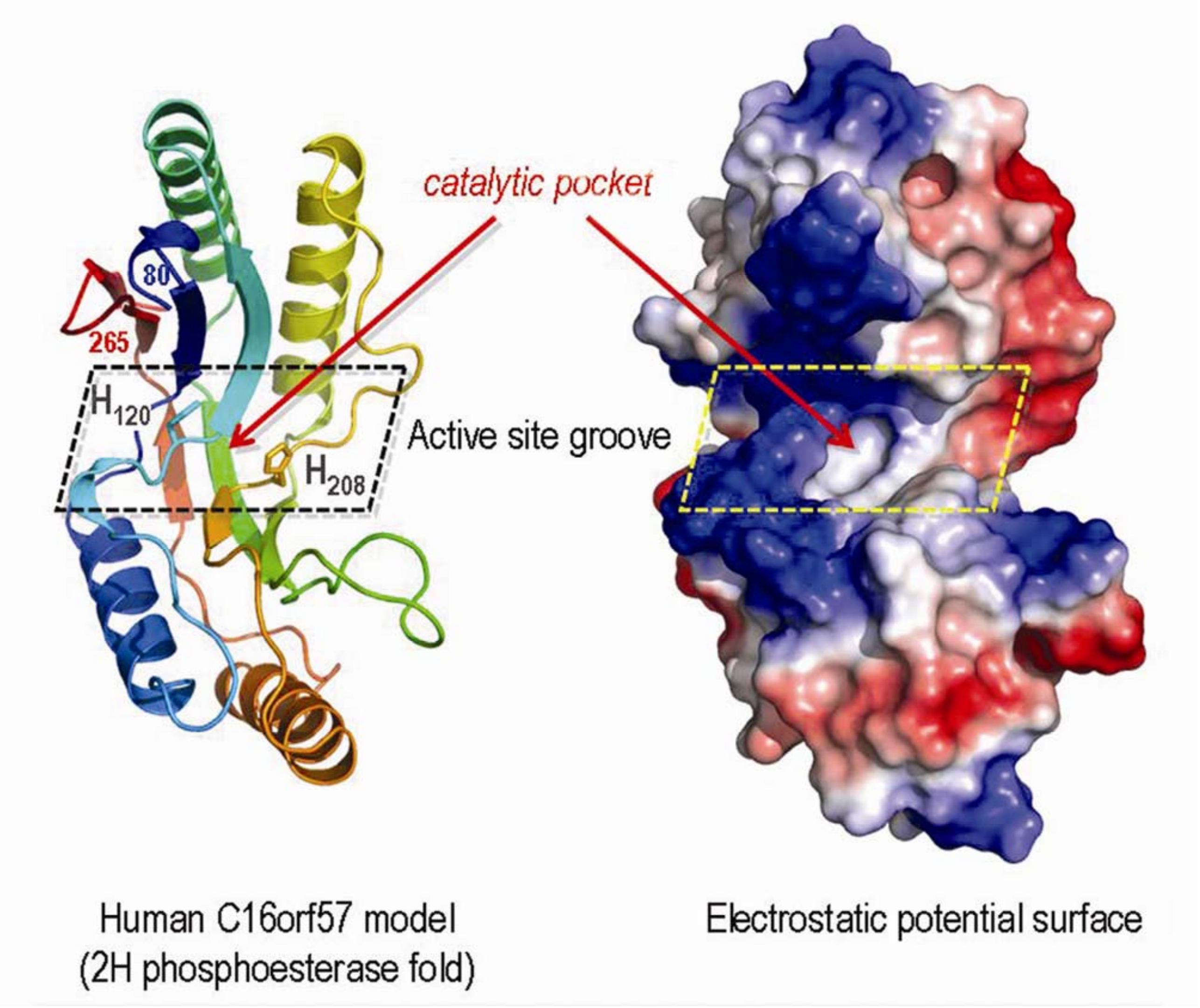

Supplement: Additional file 3 — Electrostatic potential surface of human C16orf57 predicted protein. The solvent-accessible surface of the I-TASSER-derived [21] structural model of human C16orf57 was displayed in Pymol http://www.pymol.org and coloured according to the electrostatic potential (ESP) ranging from blue (positively charged or basic) to red (negatively charged or acidic). In a top view that looks directly down at the active site groove, the corresponding ESP surface is quite negatively charged, which is similar in nature to 2H phosphoesterase structures of the RNA ligase class that interact with positively charged substrates [29,30]. [file 1750-1172-7-7-S3.PDF]
